# The Daisho Peptides Mediate *Drosophila* Defense Against a Subset of Filamentous Fungi

**DOI:** 10.3389/fimmu.2020.00009

**Published:** 2020-01-23

**Authors:** Lianne B. Cohen, Scott A. Lindsay, Yangyang Xu, Samuel J. H. Lin, Steven A. Wasserman

**Affiliations:** Section of Cell and Developmental Biology, Division of Biological Sciences, University of California, San Diego, La Jolla, CA, United States

**Keywords:** innate immunity, toll, *Drosophila*, humoral, antifungal

## Abstract

Fungal infections, widespread throughout the world, affect a broad range of life forms, including agriculturally relevant plants, humans, and insects. In defending against fungal infections, the fruit fly *Drosophila melanogaster* employs the Toll pathway to induce a large number of immune peptides. Some have been investigated, such as the antimicrobial peptides (AMPs) and Bomanins (Boms); many, however, remain uncharacterized. Here, we examine the role in innate immunity of two related peptides, Daisho1 and Daisho2 (formerly IM4 and IM14, respectively), found in hemolymph following Toll pathway activation. By generating a CRISPR/Cas9 knockout of both genes, Δ*daisho*, we find that the Daisho peptides are required for defense against a subset of filamentous fungi, including *Fusarium oxysporum*, but not other Toll-inducible pathogens, such as *Enterococcus faecalis* and *Candida glabrata*. Analysis of null alleles and transgenes revealed that the two *daisho* genes are each required for defense, although their functions partially overlap. Generating and assaying a genomic epitope-tagged Daisho2 construct, we detected interaction *in vitro* of Daisho2 peptide in hemolymph with the hyphae of *F. oxysporum*. Together, these results identify the Daisho peptides as a new class of innate immune effectors with humoral activity against a select set of filamentous fungi.

## Introduction

Fungal infections have a devastating impact on a wide range of organisms. They are destructive to agricultural plants around the world, including rice, wheat, and tomatoes (1). Additionally, fungi infect more than one million humans annually (2). Existing antifungal treatments are limited, with only one new class of drugs, echinocandins, developed in the past 15 years. Furthermore, extensive usage of limited classes of related antifungals has led to the increasingly frequent appearance of drug-resistant fungi (2). An enhanced understanding of naturally occurring antifungal defenses is thus of tremendous potential benefit.

The fruit fly *Drosophila melanogaster* is a robust model for fungal infections, replicating many features of murine fungal infections (3, 4). In the wild, flies have been found to be infected with a number of filamentous fungi, including *Beauveria, Metarhizium*, and *Fusarium* species (5, 6). In combatting these infections, flies rely on the Toll innate immune pathway (7, 8). Toll provides defense against not only filamentous fungi, but also yeasts and those Gram-positive bacteria that produce a cell wall containing Lys-type peptidoglycan (8–10). A second innate immune pathway, defined by the Imd receptor, provides defense against Gram-negative bacteria and the limited number of Gram-positive bacteria that produce a cell wall containing DAP-type peptidoglycan (11, 12).

Systemic activation of Toll signaling induces a broad set of genes first identified by microarray analysis and mass spectroscopy (13–16). Many of the induced innate immune genes are transcribed in the fly fat body, with the protein products secreted into the hemolymph. These include antimicrobial peptides (AMPs), the Bomanin peptides, and a number of uncharacterized peptides.

Although AMPs, such as the antifungal peptide Drosomycin (Drs) directly kill pathogens *in vitro* (17, 18) and are immunoprotective when ectopically expressed *in vivo* (19), recent loss-of-function studies reveal little or no requirement for AMPs in defense against fungi and Gram-positive bacteria (20). In contrast, the Bomanin family of peptides (Boms) are required for defense against both classes of pathogens (21). Boms, which are *Drosophila*-specific, are readily detected in hemolymph following Toll activation. Here we describe the functional characterization of additional immune effectors, the Daisho peptides, which appear in hemolymph following systemic infection and are required for defense against a subset of filamentous fungi.

## Materials and Methods

### Fly Husbandry and Strain Generation

Flies were raised at 25°C on cornmeal molasses agar media^1^. The *w*^1118^ strain was used as the wild type. *MyD88*^−^ flies were *MyD88*^*kra*1^, and *imd*^−^ flies were *imd*^*shadok*^.

As described in Results, the genes for the immune induced peptides IM4 and IM14 have been given the designations *daisho1* and *daisho2*, respectively. The null allele Δ*daisho*, deleting both genes, as well as the individual gene deletions, Δ*dso1* and Δ*dso2*, were generated using CRISPR/Cas9 technology, applying methods described previously (22). Pairs of guide RNAs that targeted Cas9 to delete the region 2R: 20,868,460–20,870,480 for Δ*daisho)*, 2R: 20,868,783–20,869,392 for Δ*dso1*, and 2R: 20,870,332–20,870,728 for Δ*dso2* were cloned into the pU6-BbsI-chiRNA vector (Addgene plasmid # 45946). Homology arms of ~1 kb were cloned into pHD-DsRed (Addgene plasmid # 51434). Cas9 was provided by plasmid pBS-Hsp70-Cas9 (Addgene plasmid #46294). Constructs were based on target sequences in the *w*^1118^ strain and injected into *w*^1118^. See Table S1 for primer sequences.

The FLAG epitope tag was cloned between the signal sequence and mature peptide of Dso2 in the context of the pHD-DsRed homologous repair template. This FLAG-Dso2 construct was introduced at the *dso2* genomic locus using the Δ*dso2* guide RNAs.

Plasmids expressing *dso1* or *dso2* transcripts from the *pBomS3* promoter were made using methods previously described (23). Briefly, the *BomS3* gene promoter was placed 5′ to the ORF encoding either Dso1 or Dso2. These constructs were then each integrated via ΦC31-mediated transgenesis at an *attP* landing site located at 86Fb on the *D. melanogaster* third chromosome (BDSC stock #24749). The transgenes were crossed into the Δ*dso1* and Δ*dso2* backgrounds and homozygous stocks were derived. An empty vector control was also introduced at the 86Fb *attP* landing site.

### Microbial Cultures

For survival experiments, microbes were cultured as follows. *Enterococcus faecalis* NCTC 775 (ATCC 19433) and *Enterobacter cloacae* were grown overnight at 37°C in LB media and concentrated to an OD_600_ of 10 in 20% glycerol. *Candida glabrata CBS 138* [ATCC 2001] was grown overnight in YPD media at 37°C and concentrated to an OD_600_ of 100 in PBS, 0.1% Tween. All filamentous fungi were grown on malt extract agar plates at 29°C until sporulation was observed (10–15 days). Fungal material was then strained through glass wool with sterile water to collect spores, which were concentrated in 20% glycerol and stored at −80°C before being used at the following concentrations (in spores/ml): *Aspergillus flavus* (sequenced strain): 5 × 10^9^; *A. fumigatus* AF293 (FGSC# A1100): 6 × 10^9^; *A. parasiticus* Nor-1 mutant (NRRL #6111): 3 × 10^9^; *Botrytis cinerea* (B05.10): 3 × 10^9^; *Fusarium graminearum* (NRRL #5883): 8 × 10^8^; *F. oxysporum* f. sp. *lycopersici* 4287 (FGSC #9935): 3 × 10^8^; *F. verticillioides* (FGSC #7415): 3 × 10^9^; *Neurospora crassa*: 1 × 10^9^.

For the induction of the Toll response, heat-killed *Micrococcus luteus* was prepared as previously described (23).

### Survival Assays

Groups of 20–25 adult male flies aged 2–7 days were collected and stabbed with a needle dipped in a suspension of bacteria, yeast, or fungal spores. Where needed, *MyD88*^−^ or *Bom*^Δ55*C*^ flies were used as controls immunodeficient for the Toll-mediated response. Flies infected with *E. faecalis* were incubated at 25°C; all other infected flies were incubated at 29°C. Fly deaths were recorded at least twice per day for the duration of each experiment. Any deaths that occurred within the first 6 h were set aside to exclude from the data any deaths due to traumatic injury. The experiment was repeated three times and results combined. Statistical analyses were performed using the Gehan-Breslow-Wilcoxon test.

### MALDI-TOF

After Toll induction with heat-killed *M. luteus*, flies were incubated at 29°C for 24 h, after which hemolymph was collected via capillary as previously described (23). Hemolymph in 0.1% trifluoroacetic acid/50% acetonitrile was mixed 1:1 with Universal Matrix (Sigma-Aldrich). Samples were then dried onto a Bruker MSP 96 ground steel plate. Spectra were collected from 1,500 to 10,000 m/z for linear mode, and 1,000–5,000 m/z for reflectron mode, both with positive polarization. Peptide calibration standard II (Bruker) was used as an external calibration standard. For each genotype, at least five independent samples were collected. Representative spectra are shown. Peaks were identified via corresponding m/z values from previous studies (13, 16). Spectra were visualized using R 3.3.2 and ggplot2 2.2.1 (24, 25).

### Quantitation of Pathogen Load

Pathogen load in infected flies was measured by qRT-PCR of fungal RNA (26, 27). Adult male flies, 2–7 days old, were stabbed with a needle dipped in *F. verticillioides* at 3 × 10^9^ spores/ml. Flies were then incubated at 29°C. Groups of 5–6 flies were collected at the stated times and frozen in liquid nitrogen. Total RNA was isolated with TRIzol (Ambion) and cDNA was made via SuperScript RT II (Invitrogen). *EF1A* was selected as a proxy gene for fungal load based on its stable expression (28). Measurements by qRT-PCR were performed on the iQ5 cycler (BioRad) with iQ SYBR Green Supermix (BioRad) using the primers listed below. Values were normalized to fly mRNA based on expression of the *rp49* gene.

Primers: Fv_EF1A_F1: GGCTTTCACTGACTACCCTCCTCT, Fv_EF1A_R1: ACTTCTCGACGGCCTTGATGACAC, rp49_F1: CAAGGGTATCGACAACAG, rp49_R1: CTTGTTCGATCCGTAACC.

### Peptide Gel Electrophoresis and Immunoblotting

Hemolymph samples were collected via the Zymo-Spin IC column method (23) from 30 male flies aged 2–7 days that had been induced with heat-killed *M. luteus* and incubated for 24 h at 29°C. Samples were run on a SDS-tricine, 18% separating/10% spacer/4% stacking, acrylamide gel^2^. Protein samples were then transferred to a PDVF membrane, blocked with 5% milk in TBST and stained with primary α-FLAG M2 (Sigma) (1:500) and secondary sheep α-mouse HRP (Amersham Biosciences) (1:1,000). The immunoblot was then treated with West Pico PLUS substrate (Thermo Scientific) and exposed to film.

### Peptide Hyphal Binding and Immunofluorescence

The immunostaining protocol was adapted from Luo et al. (29). *F. oxysporum* was grown in 5 ml malt extract broth from a starting concentration of 2.9 × 10^5^ spores/ml. After overnight shaking at room temperature, fungal hyphae were collected by centrifugation at 1,000 g for 10 min and resuspended in PBS. Hemolymph was collected via the Zymo-Spin IC column method (23) from 420 male flies that had been induced with heat-killed *M. luteus* 24 h prior and incubated at 29°C, yielding ~35 μl cell-free hemolymph. Next, aliquots of 200 μl hyphae and 35 μl hemolymph were shaken at room temperature for 30 min. The samples were washed three times with PBS before fixation with 4% formaldehyde for 1 h. After washing another three times with PBS, samples were blocked for 1 h with 5% BSA. Samples were then incubated with α-FLAG antibody (1:200) overnight at 4°C. After washing with PBS, samples were stained for 2 h with donkey α-mouse Alexa555 (1:400) and DAPI (1:200) and then washed and mounted on slides. Samples were imaged with a Ti2 Widefield microscope (Nikon) and analyzed with the NIS- elements software and OMERO.

## Results

### Generation of Flies Null for the *daisho* Gene Pair

Pioneering mass spectrometry experiments by Bulet et al. identified two dozen peptide IMs (immune-induced molecules) that accumulate in *Drosophila* hemolymph upon induction of the innate immune response, principally the Toll pathway (13, 16). Among these, the Bomanins have been found to play an essential role against a broad range of pathogens (21, 23) while several, including the 15 aa long IM4 and 24 aa long IM14, have unknown functions. Based on our demonstration of defensive functions for these peptides, we have renamed them Daisho1 and Daisho2, for 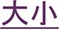 *daisho*, the Japanese term for a matched pair of samurai swords, one short and one long.

**Figure 1:**
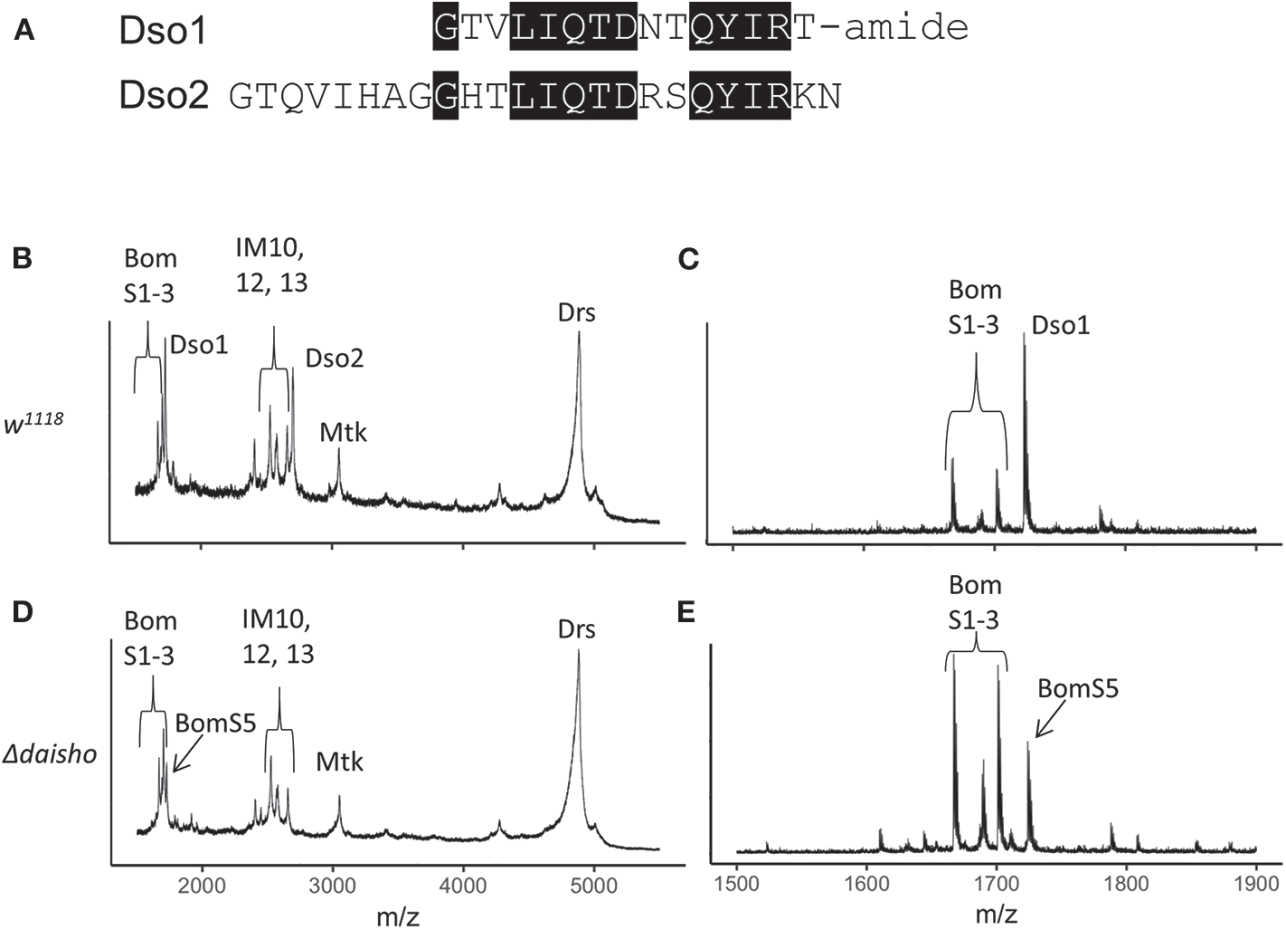
Deletion of *Drosophila daisho1* and *daisho2* gene pair. **(A)** Alignment of mature Daisho1 and Daisho2 peptide sequences. Identical residues are highlighted. **(B–E)** Mass spectrometry analysis of Toll-induced hemolymph in linear **(B,C)** and reflectron **(D,E)** mode, illustrating loss of Daisho1 (Dso1, formerly IM4) and Daisho2 (Dso2, formerly IM14) signal in Δ*daisho* deletion mutant. The Dso1 signal overlaps with the BomS5 signal, which is readily apparent in the Δ*daisho* mutant analyzed in reflectron mode. Mtk, Metchnikowin; Drs, Drosomycin.

The Daisho peptides are closely related to one another and occupy adjacent positions in the genome, where they are divergently transcribed (Figure S1). As shown in Figure 1A, the sequence of amidated mature Daisho1 (Dso1) has 67% identity with the corresponding region of the mature Daisho2 (Dso2) peptide. Like the Bomanins, the *daisho* genes are widespread among the *Drosophila* genus, but not identified elsewhere. To investigate the potential role of the *daisho* genes in innate immunity, we used CRISPR/Cas9 technology to delete both genes. The 2.0 kb deleted region includes the entire *dso1* gene, the upstream region for both genes, and the first exon of *dso2* (including the start codon). Flies homozygous for the Δ*daisho1,2* deletion, hereafter Δ*daisho*, were viable and fertile.

With the Δ*daisho* stock in hand, we carried out MALDI-TOF studies of hemolymph (Figures 1B–E). As described above, following Toll activation wild-type hemolymph displays robust expression of immune peptides, including the Daisho peptides, Bomanins, and AMPs. The signals from Dso1 and Dso2 were ablated in Δ*daisho*, as evidenced by the loss of signal at 1,722 mass/charge (m/z) (Dso1) and 2,694 m/z (Dso2). Furthermore, the spectra of induced Δ*daisho* hemolymph was wild-type for all previously identified peaks other than Dso1 and Dso2, including the Bomanins and AMPs, Metchnikowin (Mtk), and Drosomycin (Drs). The absence of Dso1 and Dso2 thus did not detectably alter the accumulation or modification of other Toll-induced peptides in the hemolymph.

In addition to previously identified peaks, Δ*daisho* hemolymph contained one previously unseen signal. The 1,724 m/z signal of this peak, readily apparent in reflectron mode, is identical to that predicted for the BomS5 amidated peptide, previously known as CG15065 (Figure 1E). This signal had not been detected previously because in the wild type it lies in the shoulder of the robust Dso1(IM4) peak. Its existence in Toll-induced hemolymph was expected, however, on the basis of microarray and RNAseq data demonstrating strong Toll-activated induction of the *BomS5* locus (15, 30).

### The *daisho* Genes Are Specifically Required for Defense Against *F. oxysporum*

We next turned to a functional assay to determine whether the absence of the Daisho peptides impaired survival following systemic infection. Because the Toll pathway responds to and protects against infection by many Gram-positive bacteria and fungi, we focused on these classes of pathogens. We stabbed adult flies with a needle dipped in a suspension of bacteria, yeast, or fungal spores and then monitored survival. We used *w*^1118^ flies as our wild-type, i.e., immunocompetent, control and *Bom*^Δ55*C*^ flies, which lack the 10-gene *Bom* cluster, as an immunodeficient control (21).

**Figure 2:**
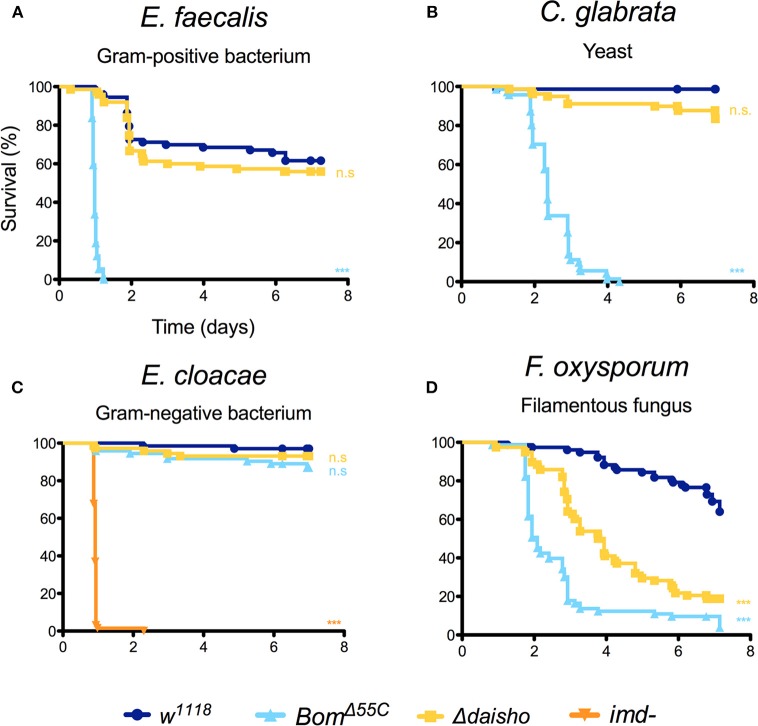
Survival of Δ*daisho* against *E. faecalis* **(A)**, *C. glabrata* **(B)**, *E. cloacae* **(C)**, and *F. oxysporum* **(D)** infection. Shown is the combination of three independent experiments for each pathogen with 20-25 flies per genotype per experiment. Survival curves were compared using the Gehan-Breslow-Wilcoxon test. Significance is shown relative to *w*^1118^ (***p < 0.0001; n.s., not significant; *p* > 0.01).

For a number of the pathogens tested, Δ*daisho* flies behaved identically to the wild type. Roughly 50% of both wild-type and Δ*daisho* flies survived 6 or more days following infection with the Gram-positive bacteria *Enterococcus faecalis*, whereas 100% of *Bom*^Δ55*C*^ flies died within 2 days (Figure 2A). Likewise, wild-type and Δ*daisho* flies survived a week or longer after infection with the yeast *Candida glabrata*, whereas *Bom*^Δ55*C*^ flies died in 4 days or fewer (Figure 2B). We also found no effect of Δ*daisho* on immune defenses mediated by the Imd pathway: wild-type, Δ*daisho*, and *Bom*^Δ55*C*^ flies all survived infection with the Gram-negative bacteria *Enterobacter cloacae*, whereas, control *imd-* flies died within 1 day (Figure 2C).

For one pathogen in the initial test set, the filamentous fungus *Fusarium oxysporum*, deletion of the *daisho* genes had a marked effect on survival (Figure 2D): 50% of flies homozygous for Δ*daisho* died within 4 days of infection. In contrast, <70% of wild-type flies survived 7 or more days post-infection. Thus, loss of the Daisho peptides disrupts defense against *F. oxysporum*, but not other tested pathogens. Interestingly, loss of the Daisho peptides did not impact survival peptides did not impact survival as severely as did loss of the Boms, which led to 50% death after 2 days, very similar to complete loss of Toll signaling (21).

### Δ*daisho* Flies Are Susceptible to Some but Not All Filamentous Fungi

**Figure 3:**
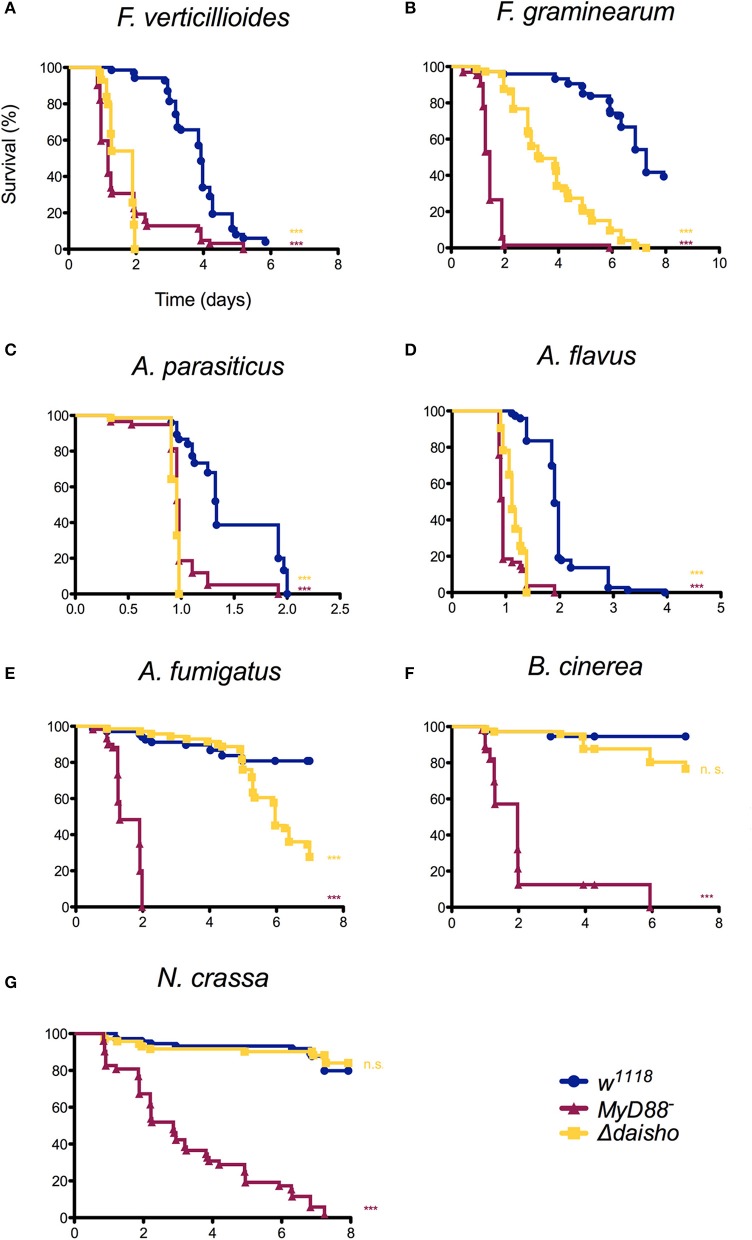
Survival of Δ*daisho* against *F. verticillioides*, **(A)**, *F. graminearum* **(B)**, *A. parasiticus* **(C)**, *A. flavus* **(D)**, *A. fumigatus* **(E)**, *B. cinerea* **(F)**, and *N. crassa* **(G)**. The combination of three independent experiments for each pathogen with 20-25 flies per genotype per experiment is shown. Survival curves were compared using the Gehan-Breslow-Wilcoxon test. Significance is shown relative to *w*^1118^ (***p > 0.0001; n.s., not significant; *p* > 0.01).

We next investigated whether the susceptibility of Δ*daisho* flies to *F. oxysporum* reflected a general susceptibility to filamentous fungi. For these studies, we focused on filamentous fungi for which flies deficient for Toll signaling, and thus for induction of Daisho1, Daisho2, and other Toll effectors, exhibit a significantly decreased survival relative to wild type (Figure 3). The control fly strains in each case were *w*^1118^ (wild type) and *kra-1* (*MyD88*^−^), a loss-of-function allele for an essential mediator of Toll signaling (31).

As shown in Figure 3, susceptibility of Δ*daisho* flies to the filamentous fungi species varied. Survival was significantly less than wild-type for *F. verticillioides* and *F. graminearum* (panels A, B)*, two Fusarium* species closely related to *F. oxysporum*. In the case of *F. graminearum*, survival of Δ*daisho* flies was intermediate between that of wild-type and *MyD88*^−^ flies, a pattern very similar to that observed with *F. oxysporum*, where Δ*daisho* survival falls between wild type and *Bom*^Δ55C^, which behaves similarly to *MyD88*^−^ (21). In contrast, Δ*daisho flies* displayed a much greater immune impairment upon infection with *F. verticillioides* than with *F. oxysporum*, dying to a comparable extent and at a similar rate as the *MyD88*^−^ control (compare Figures 2D, 3A).

Variation in survival was also observed among *Aspergillus* species. The survival curves of Δ*daisho* infected with either *A. parasiticus* or *A. flavus* largely tracked with *MyD88*^−^ (panels C, D). Upon *A. fumigatus* infection, however, Δ*daisho* flies survived at least twice as long *as MyD88*^−^ flies (Figure 3E).

For some filamentous fungi, loss of Daisho1 and Daisho2 did not affect survival. For example, 80% of wild-type and Δ*daisho* flies survived for at least 7 days after infection with *Botrytis cinerea*, whereas >50% of *MyD88*^−^ flies died after 2 days (Figure 3F). Likewise, wild-type and Δ*daisho* flies survived *Neurospora crassa* infection for 6 days or more, but 50% of *MyD88*^−^ flies died after 3 days (Figure 3G). Overall, we find that the Daisho peptides play a vital role in survival after infection with certain species of filamentous fungi, but are not important for infections with others.

### *daisho1* and *daisho2* Are Each Required for Defense

**Figure 4:**
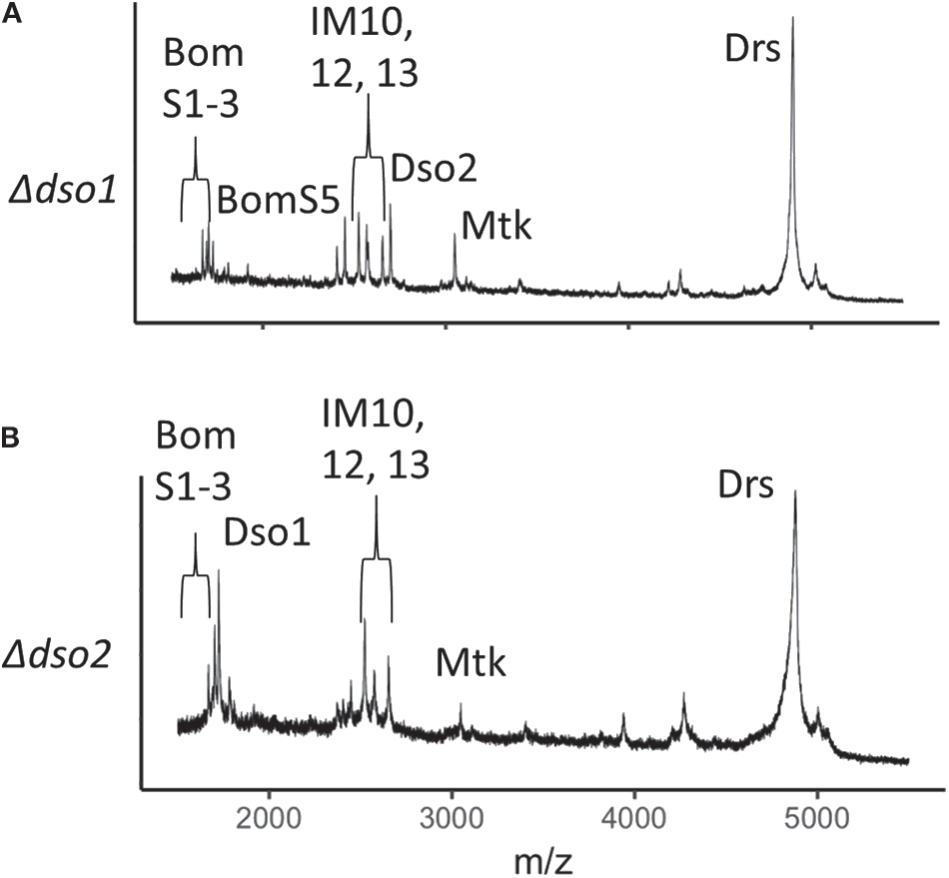
MALDI-TOF spectra for Δ*dso1* and Δ*dso2* hemolymph. **(A,B)** Mass spectrometry analysis of Toll-induced hemolymph in linear mode, highlighting loss of Dso1 **(A**) and Dso2 **(B)** in deletion mutants.

Daisho1 and Daisho2 are highly similar in sequence and expression pattern. Are they functionally redundant? To address this question, we explored the function of each individual locus. We again used CRISPR/Cas9, generating deletions that removed the entire coding sequence for either *daisho1* or *daisho2*. The 5' endpoints of each deletion were chosen to lie within 100 bp of the transcriptional start site, minimizing potential disruption of elements in the regulatory region separating the two genes (Figure S1). For both deletions, MALDI-TOF analysis of induced hemolymph confirmed loss of the deleted gene product but no other peptides, indicating that either Daisho1 or Daisho2 can be stably expressed in the absence of the other (Figure 4).

**Figure 5:**
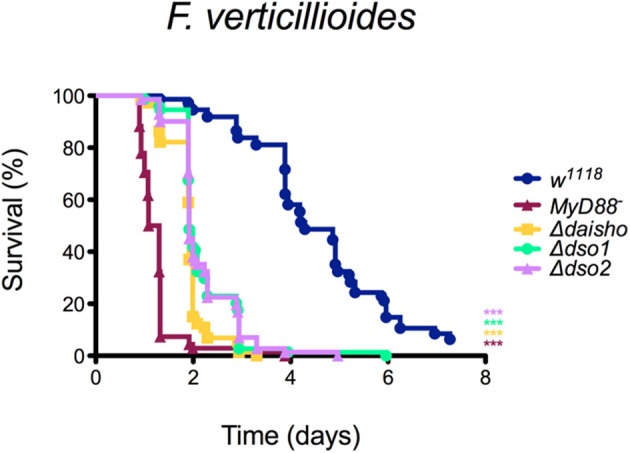
Survival of Δ*dso1* and Δ*dso2* against *F. verticillioides*. Shown is the combination of three independent experiments with 20–25 flies per genotype per experiment. Survival curves were compared using the Gehan-Breslow-Wilcoxon test. Significance is shown relative to *w*^1118^ (***p < 0.0001).

To test the effect on defense of deleting *dso1* or *dso2*, we stabbed adults with *F. verticillioides* spores, for which Δ*daisho* flies have a reduced survival. Deleting either the *dso1* or *dso2* gene resulted in susceptibility to *F. verticillioides* markedly different from wild-type and comparable to that of the double deletion (Figure 5). Thus, Daisho1 and Daisho2 each act in defense against *F. verticillioides* infection.

Since deletion of either *dso1* or *dso2* had as severe an effect on survival as the double mutant, it was possible that each gene has a specific and distinct function in antifungal defense. Alternatively, survival might depend only on total dosage for the two genes, with loss of either dropping expression below the threshold required. To distinguish between these models, we generated transgenes placing each ORF under control of *pBomS3*, shown previously to be strongly Toll-responsive promoter (23), and then assayed the transgenes for rescue of Δ*dso1* or Δ*dso2*. As shown in Table 1, *pBomS3*-driven *dso1* rescued Δ*dso1*, improving the median survival from 46 to 93 h (*p* < 0.0001). The same was true of *pBomS3*-driven *dso2* in the Δ*dso2* background (*p* < 0.0001) (see Figure S2 for full survival curves). Flies expressing the empty vector construct at the same chromosomal location did not show any increase in survival (Figure S3).

**Table 1:** Median survival in hours of *dso1* and *dso2* deletion mutations rescued by homotypic and heterotypic transgenes.

	**No transgene**	***pBomS3-dso1***	***pBomS3-dso2***
*MyD88^−^*	29	n.a.	n.a.
Δ*daisho*	46	n.a.	n.a.
Δ*dso1*	46	93	78
Δ*dso2*	46	55	93
*w^1118^*	103	n.a.	n.a.

*Data derived from Figure S2. n.a., not applicable*.

Having confirmed the activity of the two constructs, we expressed each in a background deficient for the other. *dso2* expression significantly improved survival of Δ*dso1* flies, increasing median survival from 46 to 78 h (*p* < 0.0001). Similarly, *dso1* expressed in a Δ*dso2* background improved median survival from 46 to 55 h (*p* = 0.0005). Nevertheless, rescue was incomplete. The median survival of *dso2* expressed in Δ*dso1* background (78 h) did not reach median survival of Δ*dso1* rescued with *dso1* (93 h) (n.s., *p* = 0.09). Furthermore, *dso1* did not rescue survival of Δ*dso2* (55 h) to the same level as *dso2* (93 h) (*p* < 0.0001). The data thus indicate that the two loci encode functions that are neither fully distinct nor fully redundant.

### Deleting *daisho1* and *daisho2* Results in an Elevated Pathogen Load in Infected Flies

To investigate whether Daisho1 and Daisho2 affect pathogen growth during infection, we measured fungal load after infection in Δ*daisho* and wild-type flies. After stabbing adult males with *F. verticillioides*, groups of 5–6 infected flies were collected and RNA was extracted. Fungal *EF1A* transcript levels were measured as a proxy for pathogen load and normalized to the fly reference gene *rp49*. Directly after infection (2 h), there was no significant difference between Δ*daisho* and wild-type flies by Mann-Whitney *U* test (Figure S4). By the next day, however, Δ*daisho* flies had a pathogen load roughly 10-fold greater than wild-type (*p* = 0.0317).

### FLAG-Dso2 Binds to *F. oxysporum* Hyphae

**Figure 6:**
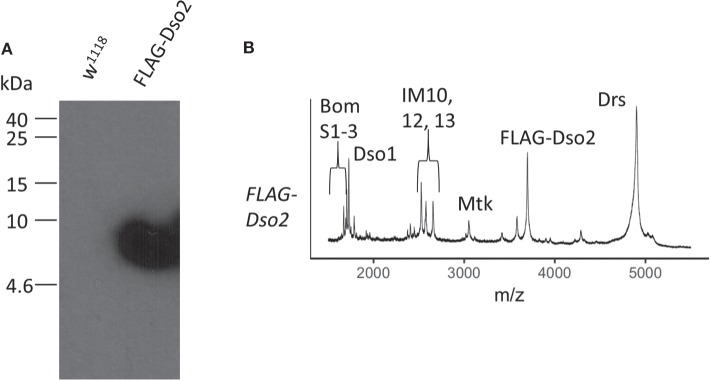
Characterization of FLAG-Dso2 gene product. **(A)** Immunoblot stained with mouse α-FLAG M2 (1:500) and sheep α-mouse HRP (1:1,000). Two μl of Toll-induced hemolymph was loaded per lane. **(B)** MALDI-TOF analysis of FLAG-Dso2 Toll-induced hemolymph in linear mode.

We next tagged Dso2, the larger of the two peptides, with the FLAG epitope, using CRISPR/Cas9 to introduce the tag at the amino-terminus of the endogenously expressed mature peptide. Immunoblot analysis of induced hemolymph from FLAG-Dso2 flies revealed a single band detectable with α-FLAG antibody (Figure 6A). MALDI-TOF analysis of hemolymph confirmed the loss of the Dso2 peak at 2,694 m/z and the appearance of a peak with an m/z ratio of 3,689, the value expected for FLAG-Dso2 (Figure 6B).

Having confirmed that FLAG-Dso2 peptide is stably expressed, we next assayed its activity in providing antifungal defense. Specifically, flies homozygous for *FLAG-dso2* at the *dso2* locus were infected with *F. verticillioides* and their survival was compared to both wild-type flies and Δ*dso2* flies. Survival of FLAG-Dso2 flies was not wild-type, but was significantly better than that of flies lacking the *dso2* gene (Figure S5). We conclude that the FLAG-Dso2 peptide is active in providing defense against *F. verticillioides* infection.

**Figure 7:**
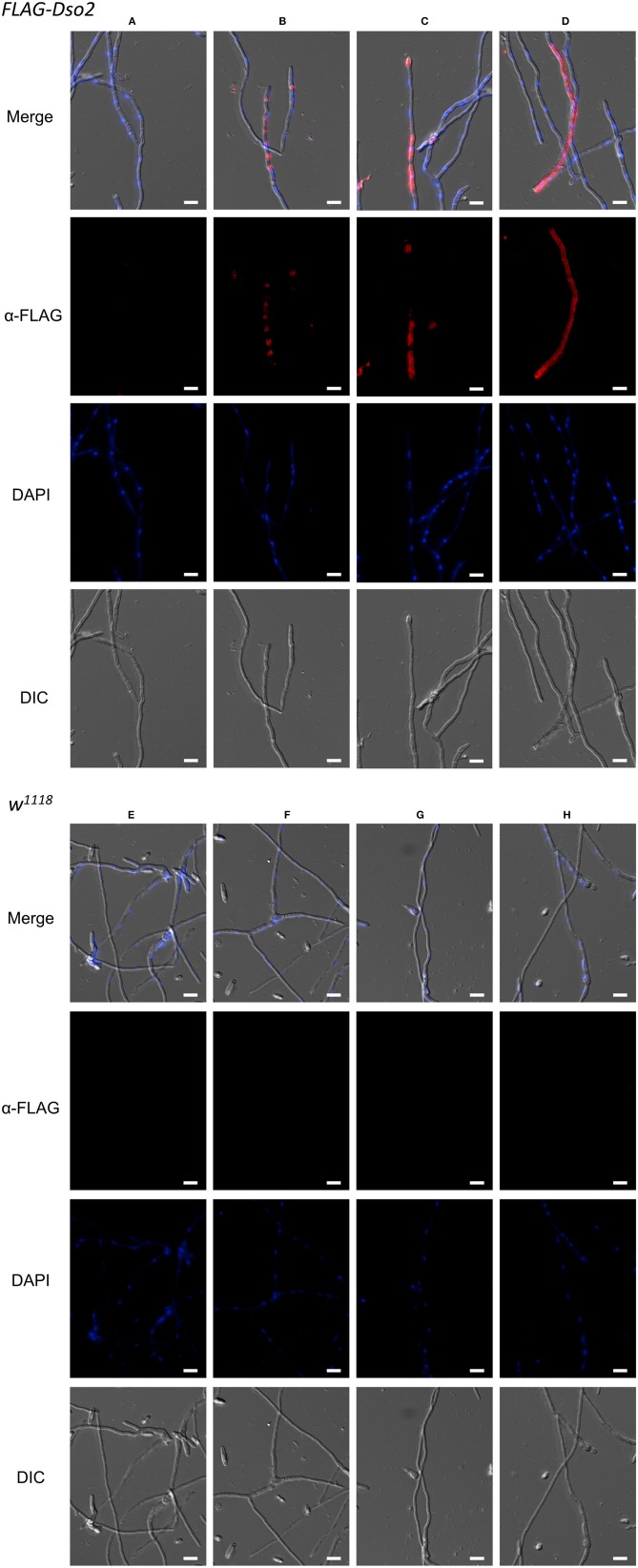
Immunofluorescence staining of *F. oxysporum* hyphae. Images showing various staining patterns of hyphae incubated with *FLAG-Dso2* **(A–D)** or *w*^1118^ hemolymph **(E–H)** and then stained with mouse α-FLAG M2 (1:200) and donkey α-mouse Alexa 555 (1:400). DAPI marks fungal DNA. Scale bar is 10 μm. Images were generated as focused images from Z-stacks.

Next, we assayed FLAG-tagged Dso2 peptide in hemolymph for its ability to bind fungus. We collected hemolymph from Toll induced flies, incubated it with hyphae from *F. oxysporum*, and fixed samples. The majority (>80%) of *F. oxysporum* hyphae had no visible signal when stained with α-FLAG antibody (Figure 7A). Among the remaining hyphae, we observed a variety of staining patterns, including, but not limited to, signals concentrated in the regions between nuclei (Figure 7B), extending across greater fractions of hyphae (Figure 7C) or spanning the length of hyphae (Figure 7D). In parallel experiments with untagged wild-type hemolymph, no signal was detected (Figures 7E–H). We conclude that Daisho2 peptide in hemolymph can bind to *F. oxysporum* hyphae.

In summary, our results demonstrate that the pair of immune-induced peptides, Daisho1 and Daisho2, mediate Toll-induced defense against specific filamentous fungi, most likely via a humoral effect on fungal hyphae.

## Discussion

### Role of the Daisho Peptides in Antifungal Defense

In this study we found that the related peptides Daisho1 and Daisho2 are required in *D. melanogaster* for defense against a subset of filamentous fungi. We have also demonstrated that the two peptides have partially overlapping functions. Survival data reveal a dependence on the overall level of Dso1 and Dso2, with each peptide able to partially compensate for the absence of the other. Furthermore, each peptide accumulates in the absence of the other.

The Daisho peptides lack known motifs of defined function. As noted previously (21), there is a similarity in size and sequence between Dso1 and Dso2 and the Bomanin peptides. There are, however noteworthy differences, including the presence of a CxxC motif in the Bomanins and the broader requirement for the Bomanins in Toll-mediated defense.

Among those fungi for which deleting *dso1* and *dso2* decreases survival, Δ*daisho* flies nevertheless often exhibit significantly greater survival than do *MyD88*^−^ or *Bom*^Δ55*C*^ flies (see e.g., *F. oxysporum* and *F. graminearum*). Thus, in contrast to the Bom effectors, which are strictly required for Toll defenses against a broad range of pathogens, the Daisho peptides appear to be required for some, but not all Toll functions and to be active against only a select group of pathogens against which Toll mounts defense.

Like the Bomanins, *dso1* and *dso2* are found only within the *Drosophila* genus. Taxonomically-restricted genes (TRGs), while often studied only sparingly, represent 10–20% of most genomes and frequently have essential functions (32). TRGs have been identified in the immune pathways of many invertebrates, including flies, mosquitoes, and cnidarians. Within immune systems they are abundant among effectors, but rare among signal transduction factors (33, 34).

### Specificity of *daisho* Genes in Antifungal Defense

In tracking survival following systemic infection, we find considerable variability with regard to which pathogens exhibit increased virulence toward *D. melanogaster* in the absence of both *daisho* genes. Categorizing the fungi against which the *daisho* genes provide defense, we detect no simple relationship to fungal phylogeny. For example, the *daisho* genes are required to defend against all the *Fusarium* species tested and some of the *Aspergillus* species, but not *Neurospora crassa*. Yet *Fusarium* and *Neurospora* are both members of the class Sordariomycetes, whereas *Aspergillus* is part of the less closely related Eurotiomycetes class (35, 36). Furthermore, Δ*daisho* flies exhibit differential susceptibility to fungi within a single genus: the Δ*daisho* deletion substantially decreases survival against *A. flavus* and *A. parasiticus*, but has a much smaller effect on survival following *A. fumigatus* infection.

Although susceptibility of Δ*daisho* flies does not track simply with fungal phylogeny, susceptibility does appear to be closely related to fungal pathogenicity. Consider four filamentous fungi that are particularly pathogenic for wild-type flies: *F. verticillioides, F. graminearum, A. flavus*, and *A. parasiticus*. Infection with any of these four pathogens kills >50% of wild-type flies within 7 days. For each of these four, Δ*daisho* greatly decreases survival. By comparison, consider filamentous fungi with low pathogenicity, e.g., *A. fumigatus, N. crassa*, and *B. cinerea*. For each, >80% of wild-type and Δ*daisho* flies survive for 7 or more days post-infection. Note that we observe this association of susceptibility with pathogenicity only among filamentous fungi: for the strongly pathogenic Gram-positive bacterium *E. faecalis*, the Δ*daisho* deletion had no effect on survival.

Although the Bomanins are strictly required for Toll humoral defenses, we have found a correlation between pathogenicity and the level of Bomanin function required to confer resistance (21). It thus appears that for both Bomanins and the *daisho* genes, pathogenicity tracks with the strength of effector function required for defense.

### Activity of Daisho Peptides

How do the Daisho peptides provide defense against filamentous fungi? One mechanism could be directly binding and killing the pathogens. Consistent with this idea, we find a modest but significant increase in pathogen load in Δ*daisho* flies. In addition, our immunofluorescence data demonstrate that Daisho2 can interact *in vitro* with at least one filamentous fungus that it targets. Antifungal peptides, such as mammalian LL-37 and plant defensin NaD1, also bind hyphae of fungal pathogens against which they are active (29, 37). The Daisho2 peptide's ability to bind fungal hyphae could indicate an antimicrobial function. Given that our assay was carried out with crude hemolymph, we cannot state whether the observed interaction of Daisho2 with hyphae is direct or is mediated by one or more unidentified hemolymph components.

The Daisho peptides might themselves interfere with pathogen growth, survival, or proliferation, or they might enable the fungicidal activity of other factors. The same is true of the Bomanins, which are required for hemolymph mediated killing of *C. glabrata*, but for which fungicidal activity of synthetic peptides has not been observed (23). Given that *daisho* genes are required for defense against only a subset of Toll and Bomanin targets, the function of Daisho1 and Daisho2 may be to meet a specific challenge posed by certain fungi to the entry or activity of antimicrobial factors. Further investigation of the Daisho peptides, as well as other hemolymph immune effectors, is likely to be informative in this regard.

## Data Availability Statement

All datasets generated for this study are included in the article/Supplementary Material.

## Author Contributions

LC and SW conceived the project and wrote the paper. LC, SAL, YX, and SJHL performed and analyzed the experiments.

### Conflict of Interest

The authors declare that the research was conducted in the absence of any commercial or financial relationships that could be construed as a potential conflict of interest.

## Footnotes

^1^http://blogs.cornell.edu/drosophila/dssc-cornmeal-recipe/

^2^https://molbio.mgh.harvard.edu/szostakweb/protocols/protein_page/index.html

## References

[1] Dean R Van Kan JAL Pretorius ZA Hammond-Kosack KE Di Pietro A Spanu PD. The Top 10 fungal pathogens in molecular plant pathology. Mol Plant Pathol. (2012) 13:414–30. 10.1111/j.1364-3703.2012.2011.00783.x 22471698 PMC6638784

[2] Janbon G Quintin J Lanternier F d'Enfert C. Studying fungal pathogens of humans and fungal infections: fungal diversity and diversity of approaches. Genes Immun. (2019) 20:403–14. 10.1038/s41435-019-0071-2 31019254

[3] Brunke S Quintin J Kasper L Jacobsen ID Richter ME Hiller E. Of mice, flies–and men? Comparing fungal infection models for large-scale screening efforts. Dis Model Mech. (2015) 8:473–86. 10.1242/dmm.019901 25786415 PMC4415897

[4] Dionne MS Schneider DS. Models of infectious diseases in the fruit fly Drosophila melanogaster. Dis Model Mech. (2008) 1:43–9. 10.1242/dmm.000307 19048052 PMC2561979

[5] Sharma L Marques G Fusarium, an entomopathogen—a myth or reality? Pathogens. (2018) 7:E93 10.3390/pathogens7040093 30487454 PMC6314043

[6] Cuthbertson AGS Audsley N. Further screening of entomopathogenic fungi and nematodes as control agents for drosophila suzukii. Insects. (2016) 7:E24. 10.3390/insects7020024 27294962 PMC4931436

[7] Imler JL. Overview of drosophila immunity: a historical perspective. Dev Comp Immunol. (2014) 42:3–15. 10.1016/j.dci.2013.08.018 24012863

[8] Valanne S Wang JH Rämet M. The Drosophila toll signaling pathway. J Immunol. (2011) 186:649–56. 10.4049/jimmunol.1002302 21209287

[9] Lemaitre B Hoffmann J. The host defense of Drosophila melanogaster. Annu Rev Immunol. (2007) 25:697–743. 10.1146/annurev.immunol.25.022106.141615 17201680

[10] Lindsay SA Wasserman SA. Conventional and non-conventional Drosophila Toll signaling. Dev Comp Immunol. (2014) 42:16–24. 10.1016/j.dci.2013.04.011 23632253 PMC3787077

[11] Georgel P Naitza S Kappler C Ferrandon D Zachary D Swimmer C. Drosophila immune deficiency (IMD) is a death domain protein that activates antibacterial defense and can promote apoptosis. Dev Cell. (2001) 1:503–14. 10.1016/S1534-5807(01)00059-4 11703941

[12] Buchon N Silverman N Cherry S. Immunity in Drosophila melanogaster–from microbial recognition to whole-organism physiology. Nat Rev Immunol. (2014) 14:796–810. 10.1038/nri3763 25421701 PMC6190593

[13] Uttenweiler-Joseph S Moniatte M Lagueux M Van Dorsselaer A Hoffmann JA Bulet P. Differential display of peptides induced during the immune response of Drosophila: a matrix-assisted laser desorption ionization time-of-flight mass spectrometry study. Proc Natl Acad Sci USA. (1998) 95:11342–7. 10.1073/pnas.95.19.11342 9736738 PMC21644

[14] De Gregorio E Spellman PT Rubin GM Lemaitre B. Genome-wide analysis of the Drosophila immune response by using oligonucleotide microarrays. Proc Natl Acad Sci USA. (2001) 98:12590–5. 10.1073/pnas.221458698 11606746 PMC60098

[15] De Gregorio E Spellman PT Tzou P Rubin GM Lemaitre B. The Toll and Imd pathways are the major regulators of the immune response in Drosophila. EMBO J. (2002) 21:2568–79. 10.1093/emboj/21.11.2568 12032070 PMC126042

[16] Levy F Rabel D Charlet M Bulet P Hoffmann JA Ehret-Sabatier L. Peptidomic and proteomic analyses of the systemic immune response of Drosophila. Biochimie. (2004) 86:607–16. 10.1016/j.biochi.2004.07.007 15556270

[17] Levashina EA Ohresser S Bulet P Reichhart J Hetru C Hoffmann JA. Metchnikowin, a novel immune-inducible proline-rich peptide from Drosophila with antibacterial and antifungal properties. Eur J Biochem. (1995) 233:694–700. 10.1111/j.1432-1033.1995.694_2.x 7588819

[18] Fehlbaum P Bulet P Michaut L Lagueux M Broekaert WF Hetru C. Insect immunity: Septic injury of drosophila induces the synthesis of a potent antifungal peptide with sequence homology to plant antifungal peptides. J Biol Chem. (1994) 269:33159–63. 7806546

[19] Tzou P Reichhart JM Lemaitre B. Constitutive expression of a single antimicrobial peptide can restore wild-type resistance to infection in immunodeficient Drosophila mutants. Proc Natl Acad Sci USA. (2002) 99:2152–7. 10.1073/pnas.042411999 11854512 PMC122334

[20] Hanson MA Dostálová A Ceroni C Poidevin M Kondo S Lemaitre B Synergy and remarkable specificity of antimicrobial peptides *in vivo* using a systematic knockout approach. Elife. (2019) 8:e44341 10.7554/eLife.44341 30803481 PMC6398976

[21] Clemmons AW Lindsay SA Wasserman SA. An effector peptide family required for Drosophila Toll-mediated immunity. PLoS Pathog. (2015) 11:e1004876. 10.1371/journal.ppat.1004876 25915418 PMC4411088

[22] Gratz SJ Ukken FP Rubinstein CD Thiede G Donohue LK Cummings AM. Highly specific and efficient CRISPR/Cas9-catalyzed homology-directed repair in Drosophila. Genetics. (2014) 196:961–71. 10.1534/genetics.113.160713 24478335 PMC3982687

[23] Lindsay SA Lin SJH Wasserman SA. Short-Form bomanins mediate humoral immunity in Drosophila. J Innate Immun. (2018) 10:306–14. 10.1159/000489831 29920489 PMC6158068

[24] Core Team R: A Language and Environment for Statistical Computing. Vienna: R Foundation for Statistical Computing (2016).

[25] Wickham H ggplot2: Elegant Graphics for Data Analysis. (2016).

[26] Troha K Buchon N. Methods for the study of innate immunity in Drosophila melanogaster. Wiley Interdiscip Rev Dev Biol. (2019) 8:e344. 10.1002/wdev.344 30993906

[27] Dostálová A Rommelaere S Poidevin M Lemaitre B. Thioester-containing proteins regulate the Toll pathway and play a role in Drosophila defence against microbial pathogens and parasitoid wasps. BMC Biol. (2017) 15:79. 10.1186/s12915-017-0408-0 28874153 PMC5584532

[28] Kim HK Yun SH Evaluation of potential reference genes for quantitative RTPCR analysis in *Fusarium graminearum* under different culture conditions. Plant Pathol J. (2011). Available online at: https://peerj.com/preprints/27537.pdf (accessed August 8, 2019).

[29] Luo XL Li JX Huang HR Duan JL Dai RX Tao RJ. LL37 inhibits aspergillus fumigatus infection via directly binding to the fungus and preventing excessive inflammation. Front Immunol. (2019) 10:283. 10.3389/fimmu.2019.00283 30842778 PMC6391356

[30] Valanne S Salminen TS Järvelä-Stölting M Vesala L Rämet M Immune-inducible non-coding RNA molecule lincRNA-IBIN connects immunity and metabolism in Drosophila melanogaster. PLoS Pathog. (2019) 15:e1007504 10.1371/journal.ppat.1007504 30633769 PMC6345493

[31] Tauszig-Delamasure S Bilak H Capovilla M Hoffmann JA Imler JL. Drosophila MyD88 is required for the response to fungal and Gram-positive bacterial infections. Nat Immunol. (2002) 3:91–7. 10.1038/ni747 11743586

[32] Khalturin K Hemmrich G Fraune S Augustin R Bosch TCG. More than just orphans: are taxonomically-restricted genes important in evolution? Trends Genet. (2009) 25:404–13. 10.1016/j.tig.2009.07.006 19716618

[33] Waterhouse RM Kriventseva EV Meister S Xi Z Alvarez KS Bartholomay LC. Evolutionary dynamics of immune-related genes and pathways in disease-vector mosquitoes. Science. (2007) 316:1738–43. 10.1126/science.1139862 17588928 PMC2042107

[34] Sackton TB Lazzaro BP Schlenke TA Evans JD Hultmark D Clark AG. Dynamic evolution of the innate immune system in Drosophila. Nat Genet. (2007) 39:1461–8. 10.1038/ng.2007.60 17987029

[35] Zhang N Castlebury LA Miller AN Huhndorf SM Schoch CL Seifert KA. An overview of the systematics of the Sordariomycetes based on a four-gene phylogeny. Mycologia. (2006) 98:1076–87. 10.3852/mycologia.98.6.1076 17486982

[36] Schoch CL Sung GH López-Giráldez F Townsend JP Miadlikowska J Hofstetter V. The ascomycota tree of life: a phylum-wide phylogeny clarifies the origin and evolution of fundamental reproductive and ecological traits. Syst Biol. (2009) 58:224–39. 10.1093/sysbio/syp020 20525580

[37] van der Weerden NL Lay FT Anderson MA. The plant defensin, NaD1, enters the cytoplasm of Fusarium oxysporum hyphae. J Biol Chem. (2008) 283:14445–52. 10.1074/jbc.M709867200 18339623

