# Supplementary figures and images for: The Daisho Peptides Mediate Drosophila Defense Against a Subset of Filamentous Fungi
Source: Front Immunol. 2020 Jan 23;11:9. doi: 10.3389/fimmu.2020.00009 (PMC6989431; doi:10.3389/fimmu.2020.00009)

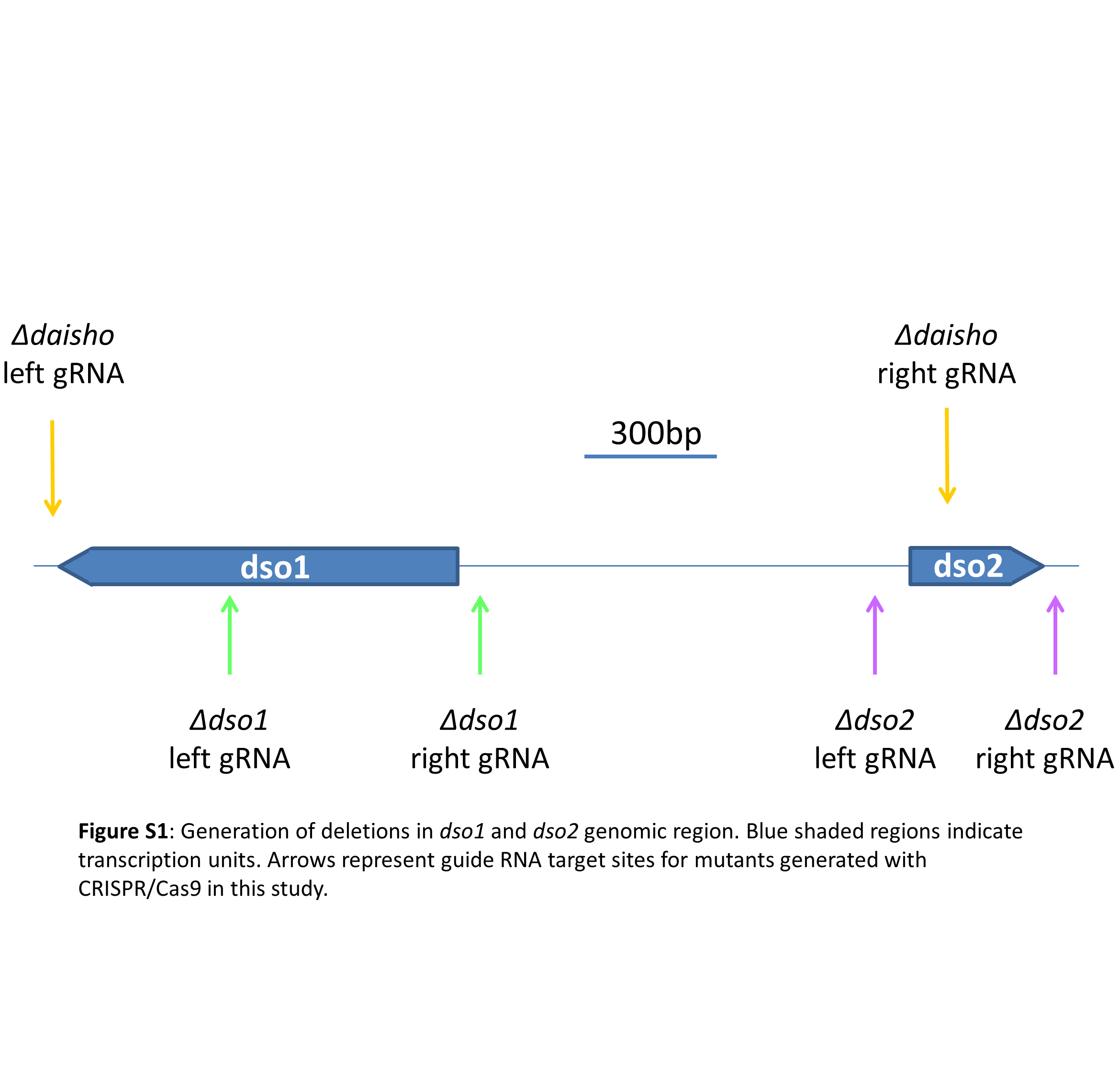

Supplement: Supplementary file 2 [file Image_1.TIF]

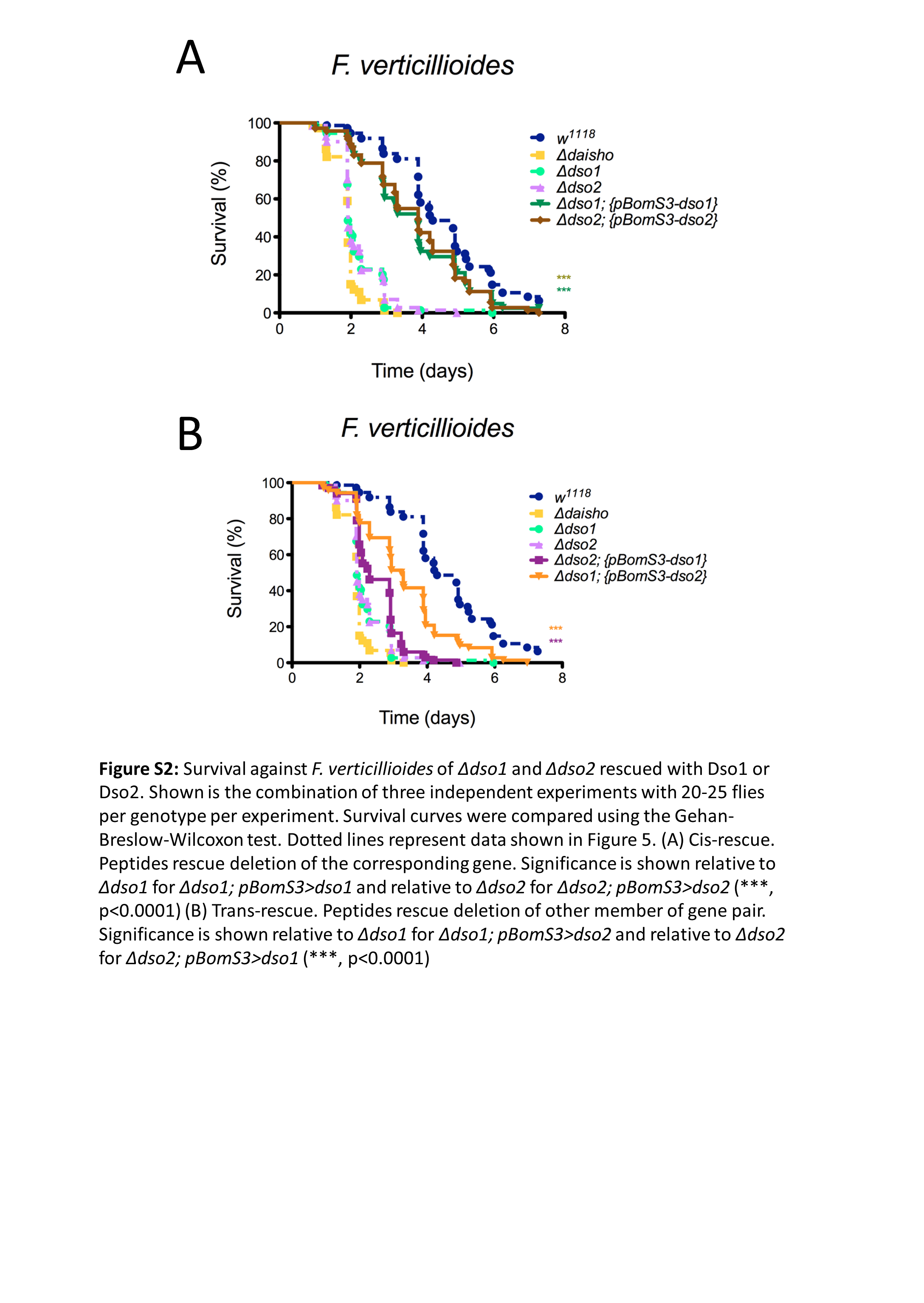

Supplement: Supplementary file 3 [file Image_2.TIF]

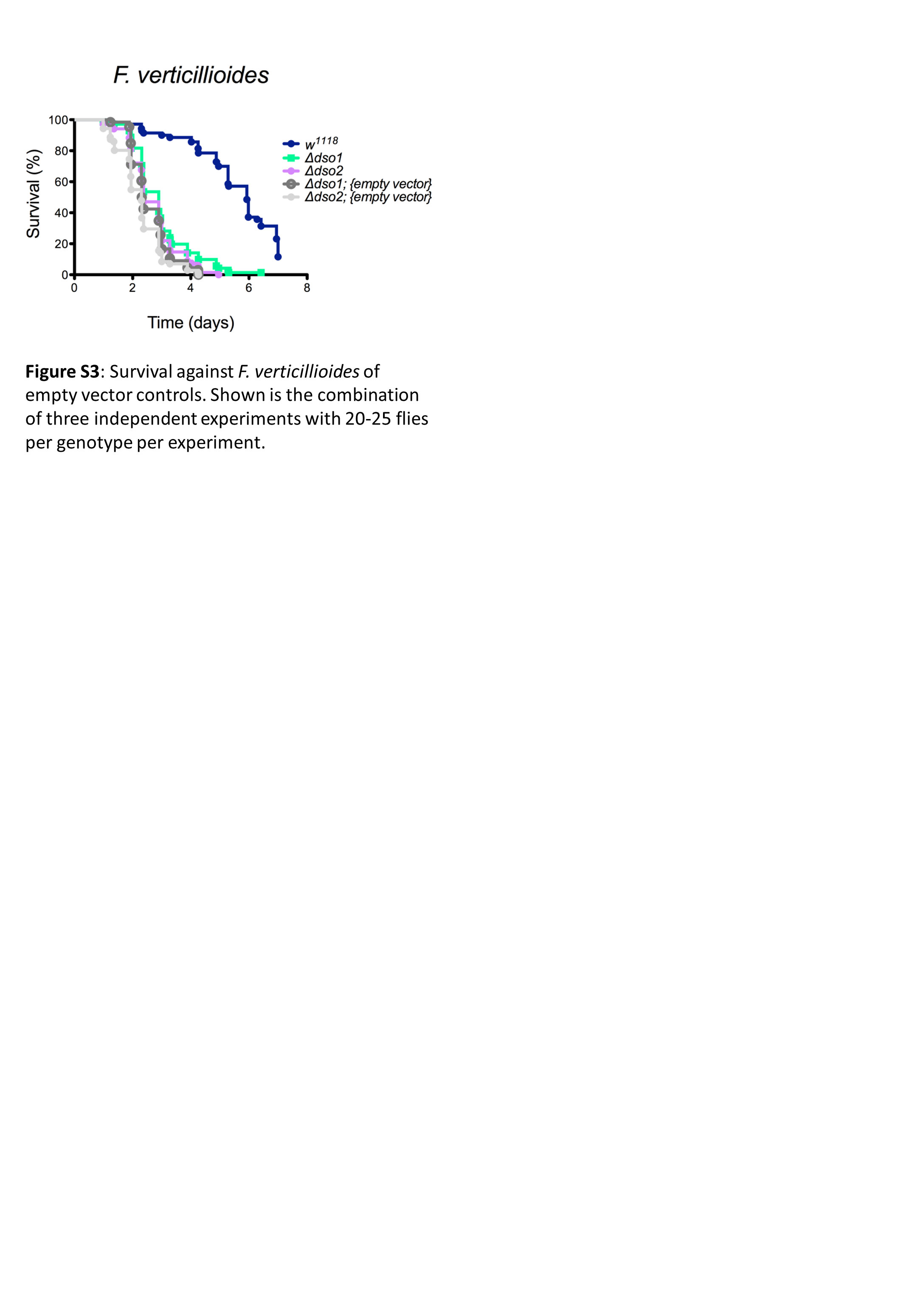

Supplement: Supplementary file 4 [file Image_3.TIF]

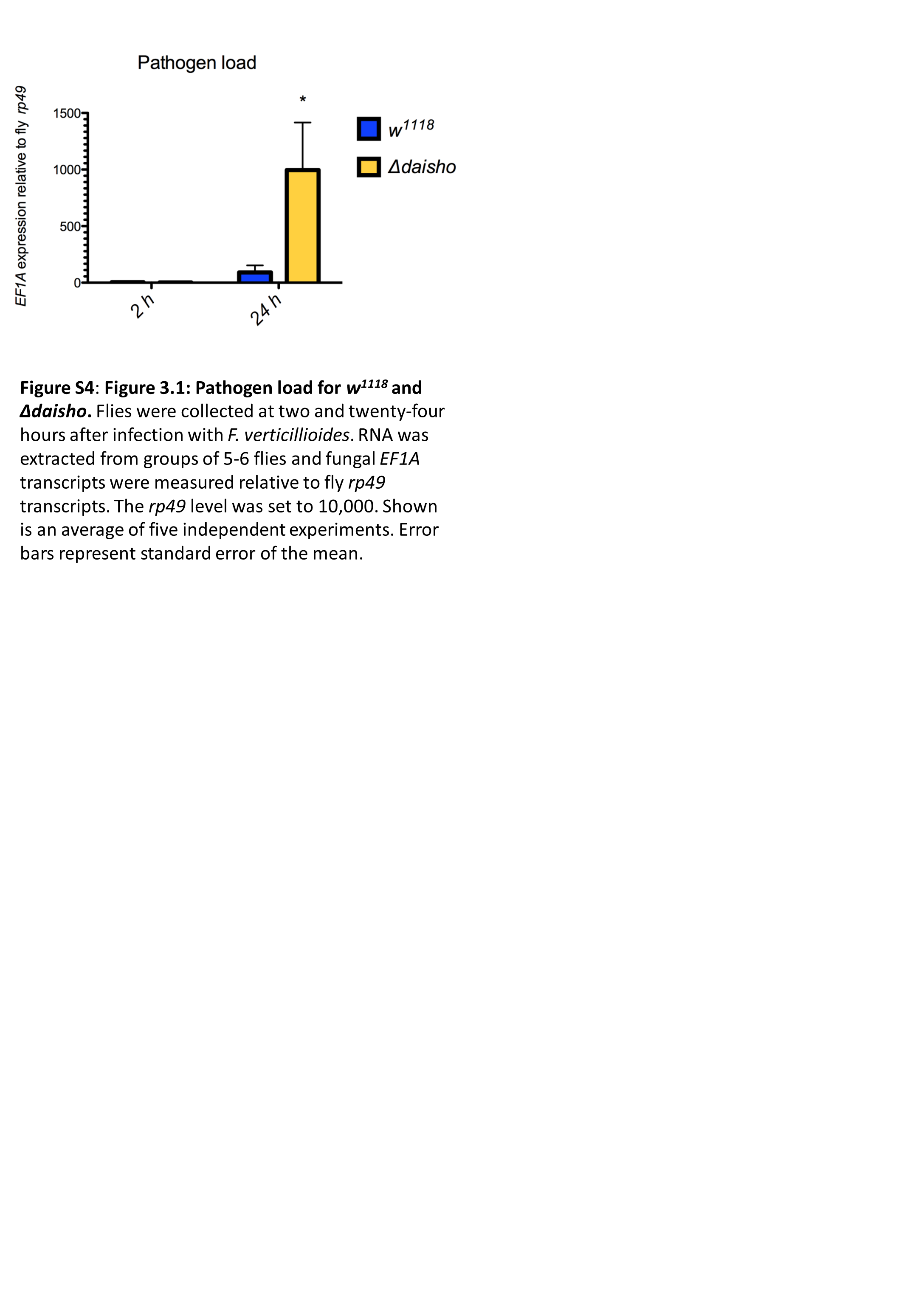

Supplement: Supplementary file 5 [file Image_4.TIF]

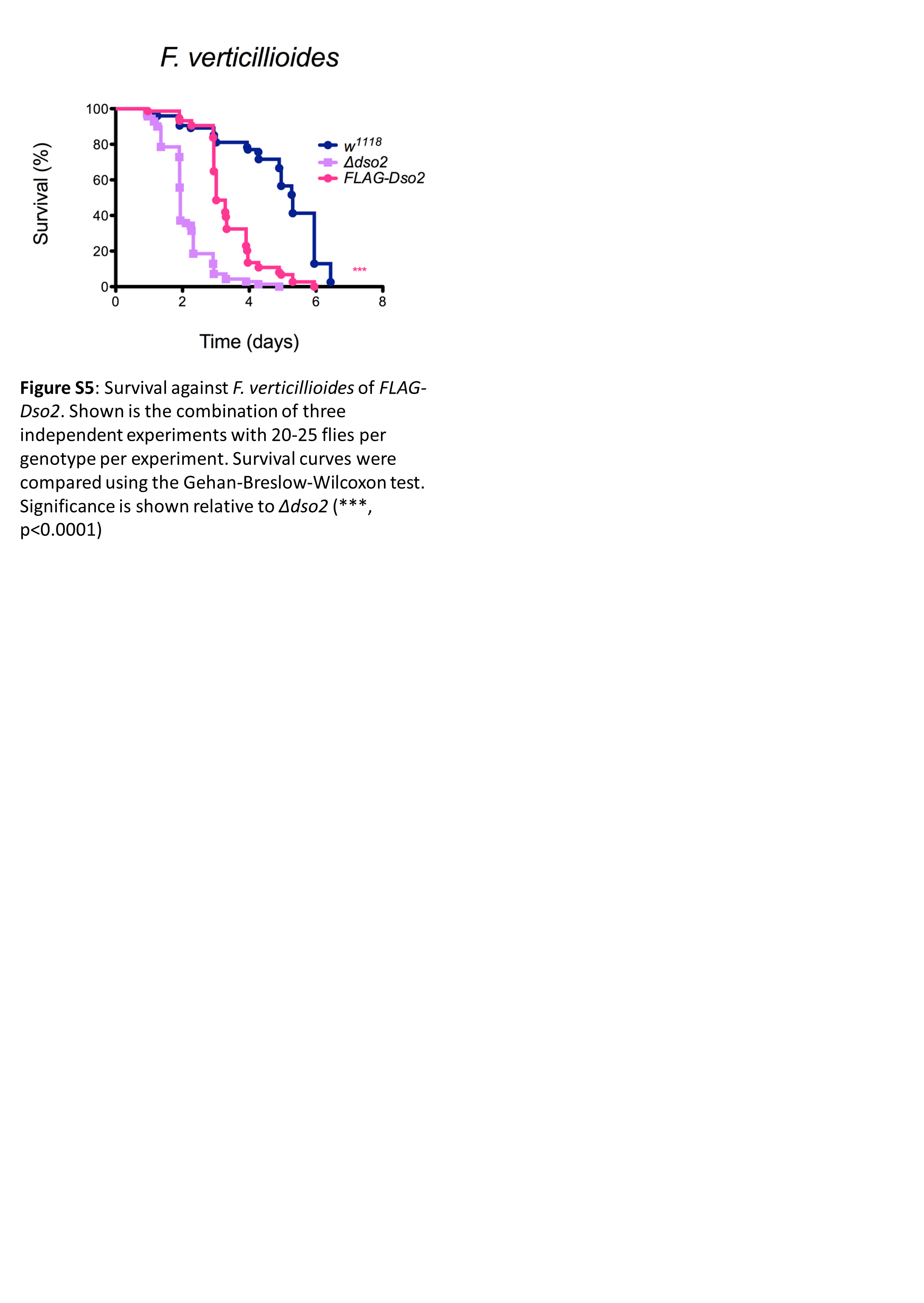

Supplement: Supplementary file 6 [file Image_5.tif]
